# Fracture With Dorsal Dislocation of the Trapezoid and Trapezium at the Scaphotrapeziotrapezoidal Joint

**DOI:** 10.1016/j.jhsg.2026.101153

**Published:** 2026-09-22

**Authors:** Aidan M. Lynch, Mihir Tandon, Owen P. Karsmarski, Joshua Baldino, Craig M. Rodner

**Affiliations:** ∗ Department of Orthopedic Surgery, University of Connecticut, Farmington, CT; † Albany Medical Center, Albany, NY

**Keywords:** Scaphotrapeziotrapezoidal dislocation, Trapezium dislocation, Trapezium fracture, Trapezoid dislocation, Trapezoid fracture

## Abstract

Scaphotrapeziotrapezoidal fracture-dislocations are extremely rare injuries, requiring early recognition and treatment. We present a case of a 54-year-old man with scaphotrapeziotrapezoidal fracture/dislocation following a motor vehicle accident, resulting in dorsal dislocation of the first and second carpal-metacarpal joints through the trapezium and trapezoid. The identification of the dislocation was delayed, and a month after the initial injury, fixation was attempted elsewhere but had redislocated at the first postoperative visit. Subsequent fixation was successful, using both radial and ulnar buried Kirschner wires. The clinical outcome was excellent with stable reduction maintained at 21 weeks postop.

Scaphotrapeziotrapezoidal (STT) dislocations are rare injuries, typically caused by direct or indirect high-energy mechanisms such as motor vehicle accidents, crush injuries, fights, or falls.^1^^,^^2^ Dislocation of either the trapezium or trapezoid itself is rare, with limited reports in the literature, and can occur either dorsally or volarly. Dorsal dislocations are more common for the trapezium, whereas volar dislocations are more common for the trapezoid.^2^ Combined dislocations are sparsely noted in the literature, with one report of a dorsal dislocation of the trapezium and a volar dislocation of the trapezoid, and another reporting a combined dorsal dislocation.^2^^,^^3^ A third author reported a case of a trapezoid dislocation with concomitant second and third carpometacarpal (CMC) joint dislocation.^4^

Treatment options for STT dislocations include open or closed fixation, with both cases described in the literature, along with arthrodesis.^2^^,^^5^ Open fixation can be performed with or without Kirschner wires (K-wires); however, recent literature favors their usage. Because of the instability of the wrist and prior operative failure, we chose an open reduction and internal fixation using buried K-wires from both the radial and ulnar sides.^1^^,^^2^^,^^6^ Of note, nonsurgical management has been suggested in the literature by Cardozo for metacarpal-trapezoid dislocations; although he notes that notable forces from the extensor carpi radialis longus and brevis and the flexor carpi radialis tendon can result in loss of the reduction.^5^

Because of the rarity of this specific injury, there is little information pertaining to the operative technique used. Rather, case reports in the literature are more descriptive about the nature of the trapezium and/or trapezoid dislocations and their causes. In this case, we want to focus on the surgical technique we used successfully for the management of this rare injury.

## Case Report

This patient is a 54-year-old man with a medical history of type 2 diabetes who was involved in a motor vehicle collision. He sustained a right dorsal fracture/dislocation of the trapezoid and trapezium at the STT joint, resulting in dorsal translation of the first and second CMC joints. His initial fracture was missed in the emergency department, and the patient presented to another orthopedic hand surgeon with symptoms of pain and decreased range of motion 1 month later. At this time, the patient was noted to have a prominence of the index CMC joint dorsally, and upon flexing the index finger, it rotated under his middle finger.

He subsequently underwent an open reduction of the dislocation and radial-sided percutaneous pinning using two 0.045-inch K-wires. The first went from the trapezium to the trapezoid, and the second went from distal to proximal from the second metacarpal base to the distal scaphoid. At the initial postop visit, X-rays demonstrated recurrence of the dorsal dislocation (Fig. 1). Hardware was removed in the office, and a CT scan was obtained revealing a dorsally translated trapezium and trapezoid, with comminuted volar rim fractures of each and the first and second CMCs dislocated dorsally through the STT fracture/dislocation (Fig. 2A–C).

**Figure 1:**
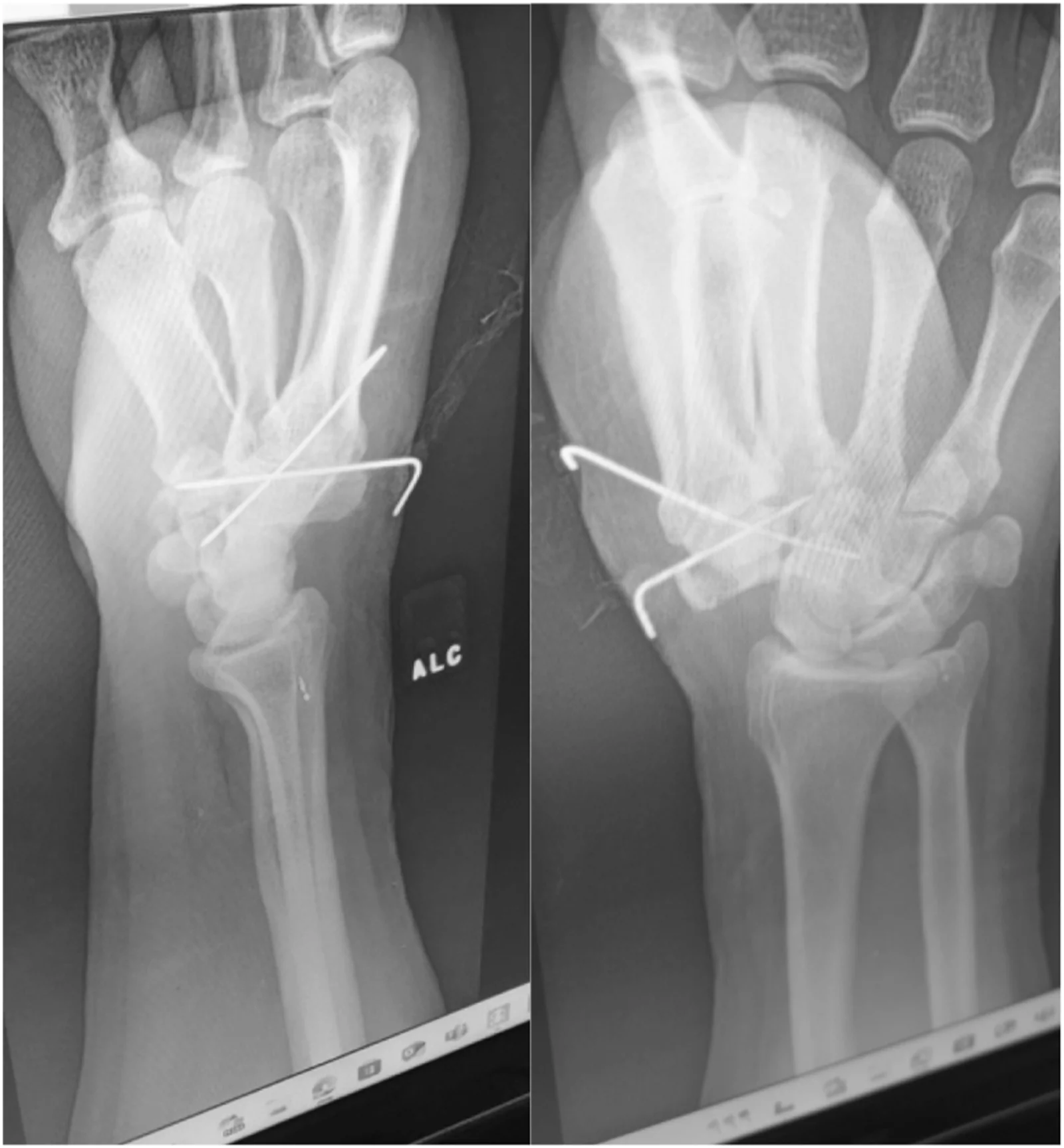
Posteroanterior (PA) and lateral plain radiographs obtained at the initial postoperative visit demonstrating redislocation of the first and second CMC joints despite the two K-wire fixation.

**Figure 2:**
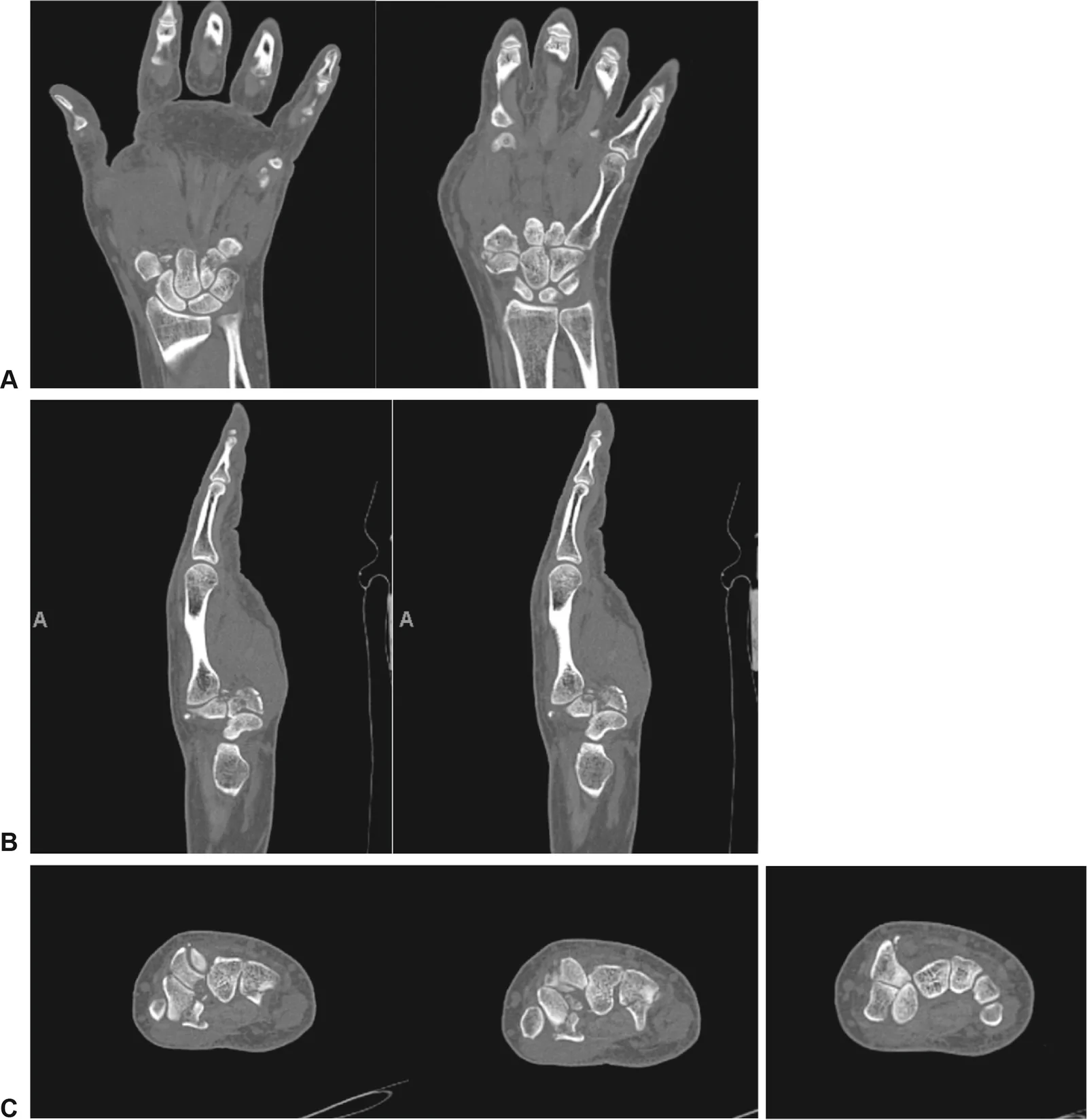
CT scan of the right hand with a **A** coronal, **B** sagittal, and **C** axial views, demonstrating comminuted fractures of the volar rim of the trapezoid and trapezium, with dorsal dislocation of the first and second metacarpal relative to the third. A nondisplaced hook of hamate fracture is also present.

The patient then presented to us, where he underwent revision open reduction and internal fixation with buried K-wires. Approach included both dorsal and volar incisions to access the dislocation from both sides as the fracture was not accessible from the dorsal side. Using a freer elevator, the trapezium and trapezoid was reduced. The reduction was then secured with five total 0.054-inch K-wires: three radial to ulnar, and two ulnar to radial (Fig. 3). The radial to ulnar K-wires stabilized the index metacarpal base in reduced position, whereas the ulnar to radial wires secured the placed to the trapezoid and trapezium to the rest of the carpus. The K-wires were cut subcutaneously to allow for longer retention given the instability of the injury and to reduce the risk of infection.

**Figure 3:**
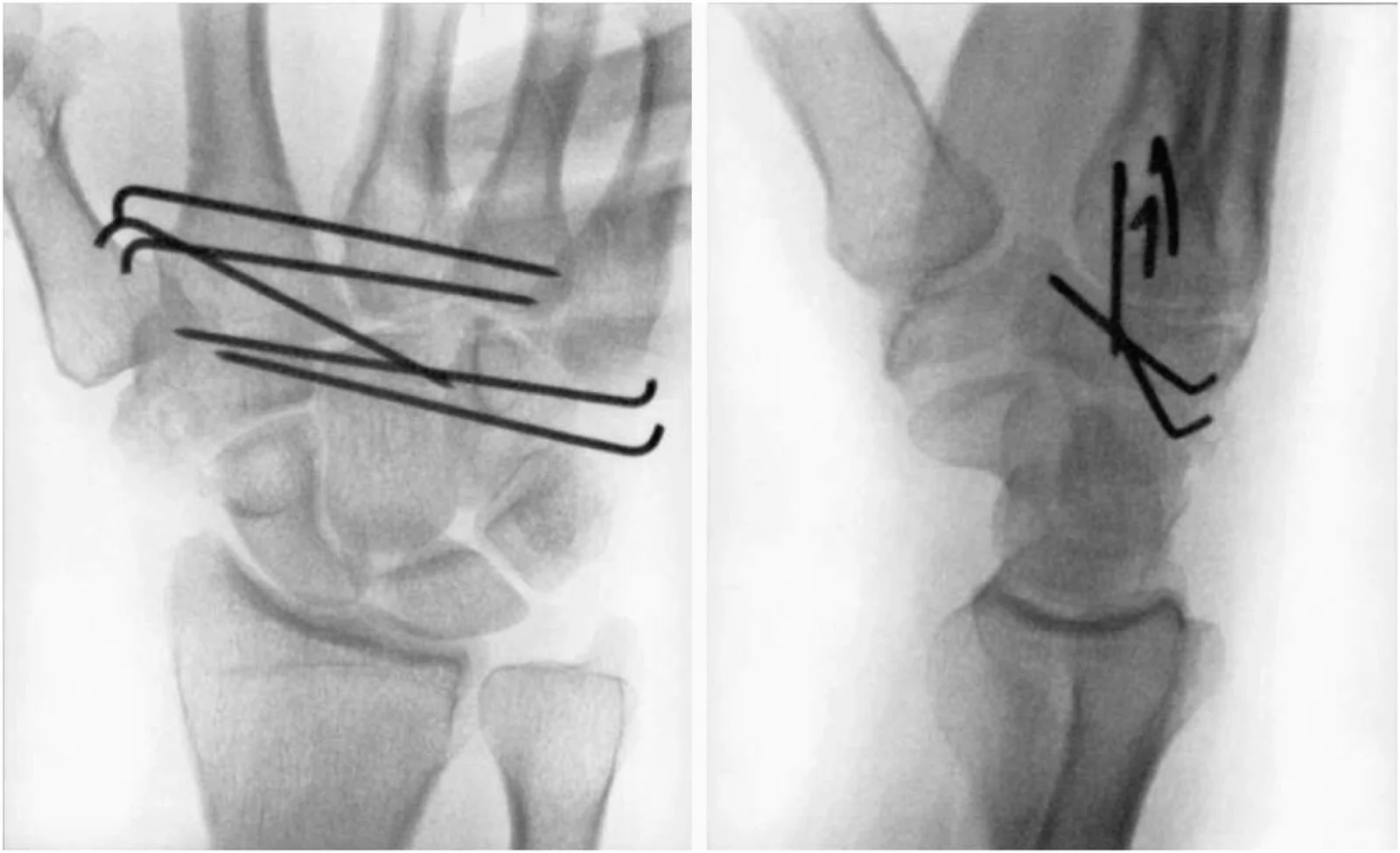
Intraoperative fluoroscopy PA and lateral views revealing revision fixation with five 0.054-inch buried K-wires.

After surgery, he was placed in a thumb spica fiberglass cast with the interphalangeal joint free, and sutures were removed on postoperative day 8. At this visit, his fingers had excellent range of motion, no malrotation, and the two incisions were pristine.

The plan was to maintain the buried pins for approximately 3 months. At 1 and 2 months after surgery, cast changes were performed and pin sites checked. At 2 months postop, he was noted to have a radial prominence of one of the pins causing discomfort underneath his skin. He was taken to the operating room, and under local anesthesia this pin was removed (Fig. 4).

**Figure 4:**
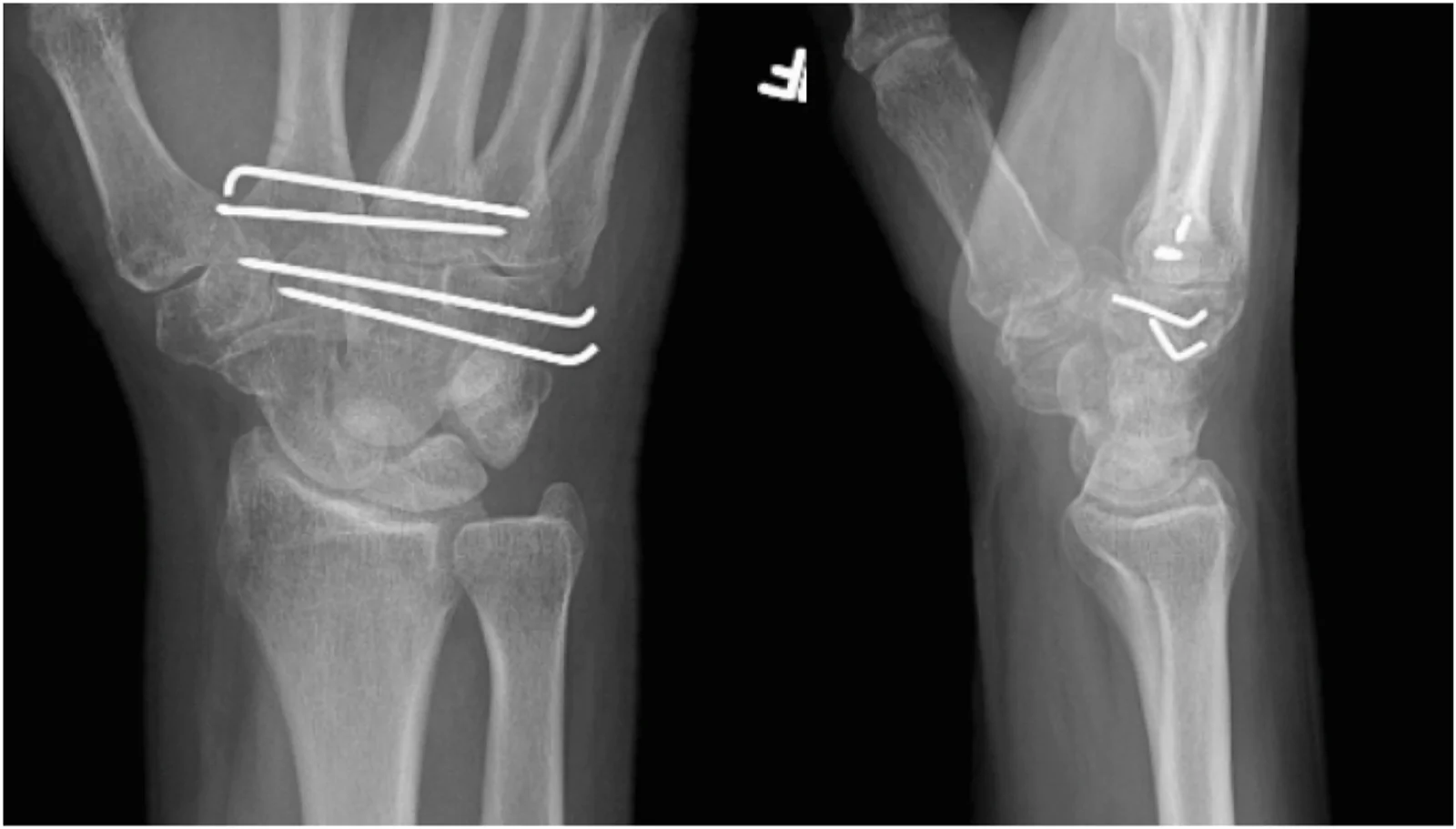
Anteroposterior (AP) and lateral plain radiographs at 2 month after surgery after removing one of the radial K-wires, which had become prominent.

Four months after his initial surgery, the patient was brought to the operating room to remove the remaining four K-wires. After pin removal, he continued with excellent digital range of motion, no pain, no malrotation, and X-rays revealed adequate, albeit not perfect, alignment (Fig. 5).

**Figure 5:**
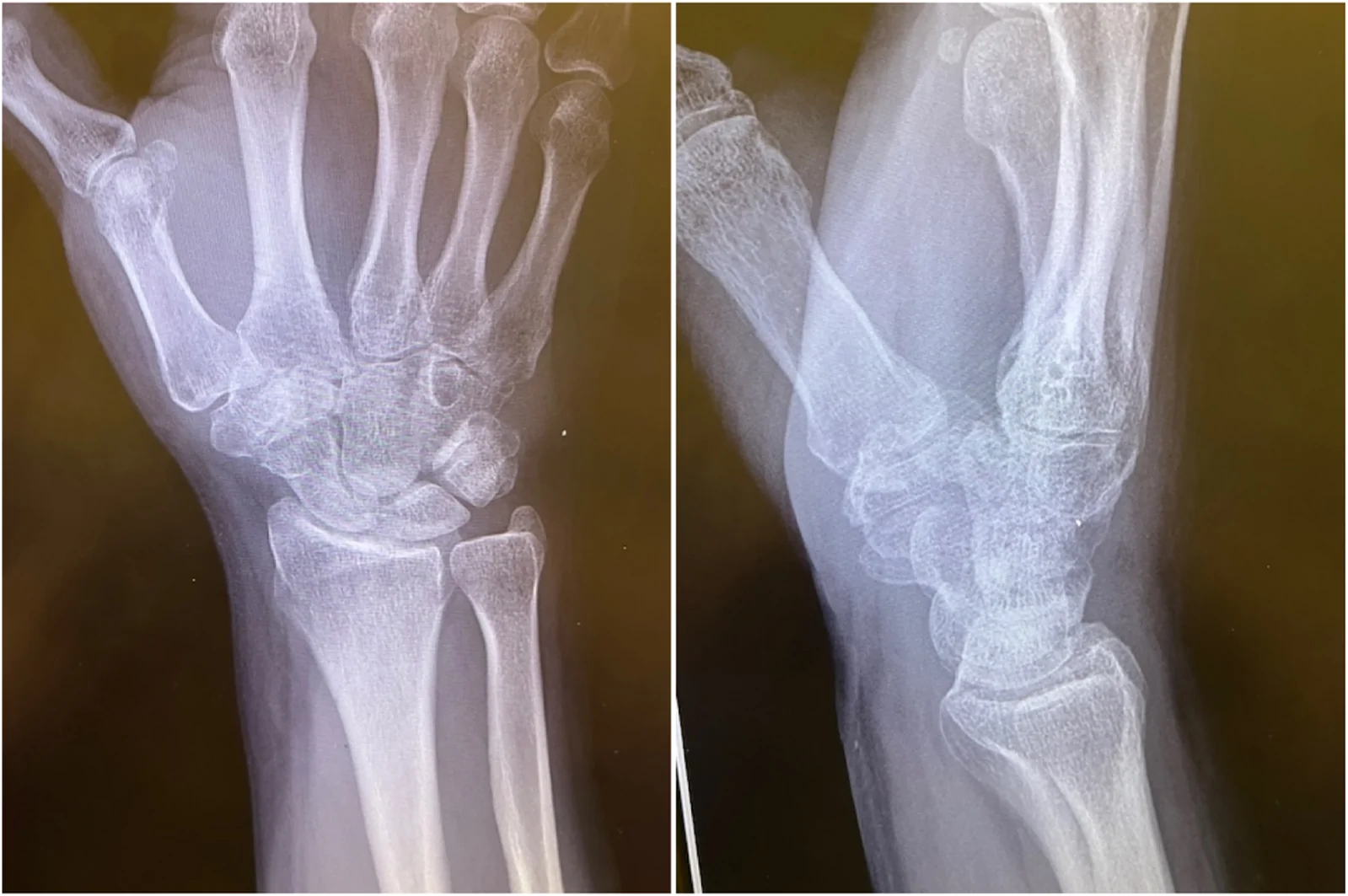
AP and lateral radiographs 4 months after revision operation with K-wires now removed.

## Discussion

STT fracture-dislocations are rare and therefore not frequently studied.^1^ Typical treatment for carpal and metacarpal fracture-dislocations includes prompt reduction, usually in the emergency department. Surgical fixation with pinning is often necessary.^7^ In this case, the carpal pinning performed initially was unsuccessful likely because of the fact that this unusual fracture-dislocation pattern was poorly understood without preoperative CT scan. We aimed that this case can severe as a helpful tool for surgeons in the future who come across this uncommon fracture-dislocation pattern.

A few pearls can be taken from this case. First, it is possible that if this injury was identified as an STT fracture-dislocation at the initial emergency department visit, this could have been closed reduced with the potential to avoid surgical intervention or perhaps be treated with percutaneous K-wire fixation. Delays in diagnosis and treatment, as in this case, can make it more difficult for reduction and fixation, especially when combined with failed prior reduction.^8^ Therefore, a major point of this case is that prompt recognition and diagnosis of this injury can make treatment much more successful. Second, stout K-wire fixation, in this case from both radial and ulnar directions, may be helpful to prevent loss of reduction. Burying K-wires subcutaneously allows the pins to stay in longer, which was helpful in this case.

In summary, we present the case of a 54-year-old man who experienced a dorsal thumb and index CMC dislocation with fracture of the trapezium and trapezoid following a motor vehicle accident. After initial operative management failed, we were successfully able to reduce the dislocation with revision surgery, resulting in a satisfactory clinical outcome at 5 months. Written informed consent was obtained from the patient for publication of this case report.

## Ethics declaration

Written informed consent to take part in the study and to publish the article has been obtained from all participants or their legal representatives. The privacy rights of participants have been observed.

This study was performed in compliance with relevant laws, regulatory frameworks and guidelines where the research took place. Ethics committee approval was not required under relevant laws and institutional guidelines. All relevant institutional guidelines were followed, with a single case report not requiring ethical review

This research follows the CARE guidelines and the CARE checklist.

## Conflicts of Interest

No benefits in any form have been received or will be received related directly to this article.
